# ErbB Family Signalling: A Paradigm for Oncogene Addiction and Personalized Oncology

**DOI:** 10.3390/cancers9040033

**Published:** 2017-04-12

**Authors:** Nico Jacobi, Rita Seeboeck, Elisabeth Hofmann, Andreas Eger

**Affiliations:** 1 Research Institute for Applied Bioanalytics and Drug Development, IMC University of Applied Sciences Krems, Krems an der Donau 3500, Austria; nico.jacobi@fh-krems.ac.at; 2 Institute of Medical and Pharmaceutical Biotechnology, IMC University of Applied Sciences Krems, Krems an der Donau 3500, Austria; rita.seeboeck@fh-krems.ac.at (R.S.); elisabeth.hofmann@fh-krems.ac.at (E.H.)

**Keywords:** ErbB family, oncogene addiction, synthetic lethality, drug discovery, tumour modeling, 3D cell culture, personalized medicine, precision therapy

## Abstract

ErbB family members represent important biomarkers and drug targets for modern precision therapy. They have gained considerable importance as paradigms for oncoprotein addiction and personalized medicine. This review summarizes the current understanding of ErbB proteins in cell signalling and cancer and describes the molecular rationale of prominent cases of ErbB oncoprotein addiction in different cancer types. In addition, we have highlighted experimental technologies for the development of innovative cancer cell models that accurately predicted clinical ErbB drug efficacies. In the future, such cancer models might facilitate the identification and validation of physiologically relevant novel forms of oncoprotein and non-oncoprotein addiction or synthetic lethality. The identification of genotype-drug response relationships will further advance personalized oncology and improve drug efficacy in the clinic. Finally, we review the most important drugs targeting ErbB family members that are under investigation in clinical trials or that made their way already into clinical routine. Taken together, the functional characterization of ErbB oncoproteins have significantly increased our knowledge on predictive biomarkers, oncoprotein addiction and patient stratification and treatment.

## 1. Signal Transduction of ErbB Receptor Tyrosine Kinases

The ErbB receptor tyrosine kinase family consists of four cell surface receptors, ErbB1/EGFR/HER1, ErbB2/HER2, ErbB3/HER3 and ErbB4/HER4 [1]. Under normal physiological conditions, ErbB receptor activation is controlled by spatial and temporal expression of their ligands [2]. Extracellular binding of ligands to their cognate receptors induces the formation of active homo- or heterodimers [3]. Seven growth factors are known to bind EGFR i.e., epidermal growth factor (EGF), epigen (EPG), transforming growth factor alpha (TGFA), amphiregulin (AREG), betacellulin (BTC), heparin binding epidermal growth factor (HB-EGF), and epiregulin (EPR). Two ligands selectively bind to ErbB3, Neuregulin (Nrg 1 and 2) and seven ligands interact with ErbB4 (BTC, HB-EGF, EPR, Nrg1-4). ErbB2 lacks a ligand binding domain and can be activated by heterodimerization with other ErbB proteins [4]. In addition, there are numerous reports showing that ErbB2 can also homodimerize and function as an active receptor and oncogenic driver when being a homodimer [5,6,7]. ErbB3 contains no functional kinase domain, but rather displays several tyrosine phosphorylation sites that provide binding sites for signalling proteins that mediate activation of downstream effector molecules such as the Akt/PKB pathway [8]. In general, ligand-induced receptor homo- or heterodimerization triggers cross-autophosphorylation and the assembly of diverse signalling molecules at the sites of the receptor dimer and activation of downstream effector circuits [1,9]. Downstream signalling networks controlled by ErbB activation consist of several interconnected and overlapping modules [1,2,4,10]. The ErbB effectors include the PI3K-Akt-mTOR pathway, the RAS-RAF-MEK-ERK pathway and the phospholipase C gamma (PLCγ) pathway [1]. These signalling cascades regulate a vast variety of physiological events including cell proliferation, apoptosis, angiogenesis, cell adhesion and motility, embryonic development, and organogenesis [10,11,12]. Particularly, EGFR and ErbB2 proteins hyperactivate theses pathways in a broad range of cancers [13,14,15,16]. The evolutionary conserved PI3K-Akt-mTOR cascade is strongly activated by ErbB3 and regulates survival, growth, and proliferation [8,17]. mTOR is crucial for the development of different human malignancies such as brain, breast, colon, liver, lung, ovary and stomach cancer [18]. Akt phosphorylates and inhibits the tumour suppressor TSC2 and thereby indirectly activates RHEB, which in turn is a positive regulator of mTOR [19]. mTOR stimulates in a nutrient and energy-sensing manner the canonical mRNA translation via activation of S6 kinase 1 and suppression of 4E-BP1 [20]. However, there is accumulating evidence that mTOR is also activated independently from ErbB receptors [20]. The importance of mTOR for tumour cell growth is now widely accepted and several agents are available or under investigation that selectively target mTOR [20]. Examples of such drugs are the rapamycin derivatives or analogs (rapalogs) RAD001, BEZ235, CCI-779 and INK-128, which are putative candidates for combination therapies with ErbB receptor inhibitors [21].

A second major signalling pathway induced by ErbB family members is the RAS-MAP-kinase pathway involving RAF, MEK and ERK. The pathway contributes to cell survival, cell growth and proliferation [22,23,24]. In this regard, the small GTPase RAS acts as a central signalling node that can activate many different downstream effector proteins [25,26,27]. RAS and particularly the subtype KRAS was one of the first molecular biomarkers used for predictive diagnostics in personalized oncology. The clinical need for KRAS mutation testing is largely associated with the use of anti-EGFR antibody therapy for patients with advanced colorectal or lung cancer [28]. There is a high frequency of KRAS mutations in colon tumours that can cause resistance to EGFR inhibitors [29]. Mutations in KRAS mainly occur in codons 12 and 13 impairing the intrinsic GTPase activity of KRAS. As a result, KRAS gets locked in a GTP-bound, active state, and constitutively triggers downstream signalling events, irrespective of upstream EGFR activity [30,31,32]. MAPK signalling results in the activation of the dimeric AP1 transcription factor composed of c-JUN and c-FOS. AP1 promotes tumourigenesis at different levels including epithelial to mesenchymal transition and cancer cell invasion and metastasis, angiogenesis, and cell proliferation and survival [33,34,35,36,37,38,39]. Furthermore, ErbB signalling can activate various other effector molecules such as PLCγ, STATs and SRC [9,40,41,42,43,44,45].

The proto-oncogene c-MET is another tyrosine kinase receptor acting independently but redundantly to ErbB family members. It is activated by hepatocyte growth factor/Scatter factor (HGF) and, similar to ErbB signalling, activates PI3K, MAP-kinase and STAT signalling [46,47,48,49,50]. The diverse array of intracellular signalling networks initiated by ErbB proteins is driving tumour progression in almost all solid cancers in humans. Hence, a new generation of drugs that selectively target the ErbB oncoproteins has demonstrated impressive therapeutic efficacy in the clinic [1,3,51].

## 2. ErbB Proteins and Oncogene Addiction

A decade has elapsed since the concept of oncogene addiction has first emerged [52]. It postulates, that despite the vast number of genetic and epigenetic changes in cancer cells, some tumours rely on the activity of a single dominant oncogene for growth and survival. Inhibition of the hyperactive oncoprotein is sufficient to halt the neoplastic growth, and cause differentiation or death of cancer cells [53]. Hence, oncogene addiction is generally considered to be the Achilles’ heel of cancer. In addition to oncogenic drivers, tumour cells may also evolve a dependency on cellular switches that are interconnected with oncogenic drivers, but do not work as oncoproteins on their own, irrespective of their mutational status. This phenomenon is commonly known as non-oncogene addiction [54,55]. Finding and targeting the critical driver molecules is a primary goal of present precision medicine [54,56,57,58]. Successful targeting and inactivation of specific driver proteins would cause a systemic failure in tumour cell physiology. However, in many cases the inactivation of a single oncoprotein is not sufficient to kill the cancer cells. Here, synthetic lethality provides a conceptual framework for the development of cancer-specific cytotoxic agents. Two genes are synthetic lethal if the deficiency of either alone is compatible with viability but the deficiencies of both leads to death. So, targeting a gene that is synthetic lethal should primarily kill cancer cells and spare the normal tissues [58,59,60].

In several tumour entities, ErbB family members have been found to be essential for cancer cell proliferation and survival [51,61,62,63,64,65]. The tumours exhibited unique expression and mutation profiles of ErbB genes, all of which had a specific impact on cancer cell differentiation, proliferation, migration, and survival. In many cases the survival of the cancer cells was strictly dependent on the mutant or overexpressed ErbB family member. The inhibition of specific ErbB proteins with low molecular weight tyrosine kinase inhibitors (TKI) or antibodies was often sufficient to cause cancer cell death [66,67]. On the contrary, certain mutations of ErbB proteins conferred resistance to treatment [48,54,68,69,70,71,72,73].

EGFR has four mutational hotspots within its tyrosine kinase domain in exons 18, 19, 20 and 21. As best described in lung cancer, single nucleotide mutations as well as deletions and insertions are associated with increased drug sensitivity (i.e., G719m, E709m, L861Q, L858R, exon 19 deletions and/or insertions) [74]. Specific mutations elevate EGFR activity, often by increasing the binding affinities for dimerization or ATP interaction [75]. In such cases, the treatments with selective drugs such as gefitinib and erlotinib have shown impressive clinical efficacies [76,77,78,79]. On the other hand, the most important alteration that has been frequently associated with acquired resistance is the point mutation T790M in exon 20 [68,72,73,80]. It occurs first and foremost in advanced tumours that have lost normal regulatory feedback circuits and use mutated EGFR to constitutively drive proliferation and survival [68]. Effective targeting of T790M mutated EGFR has been a research challenge of the last decade. With the development of osimertinib, a powerful precision drug is now available [81]. A doubling of progression free survival time from 4.2 to 8.2 months could be achieved in lung cancer patients positive for the T790M mutation [81,82]. Also mutations in the ectodomain of the EGFR can have a strong impact on cancer progression. In glioblastoma multiforme a characteristic deletion of 267 amino acids is often detected in the extracellular domain (EGFRvIII) [83]. The mutated receptor is unable to bind to ligand and yet constitutively activates mitogenic, anti-apoptotic and pro-invasive signalling pathways. The deletion also alters internalisation and degradation of the EGFR. The lack of expression of EGFRvIII in normal tissue makes it a first-rate drug target for precision medicine [83].

ErbB2 has been intensively studied in breast cancer and is known to be overexpressed in other cancer types as well, including urinary bladder, lung, digestive tract, endometrial and cervical cancer [84]. The ErbB2 oncogene is located on chromosome 17q12. Gene amplification is the primary mode of ErbB2 receptor overexpression and is a major driver of tumour development and progression in a subset of breast cancers. An ErbB2 amplification occurs in about 15–20% of breast cancers [15,84,85]. Metastatic ErbB2-positive breast cancer correlates with increased aggressiveness, poor prognosis, and short overall survival time [86]. The overexpressed receptor represents a paradigm for oncoprotein addiction, and with that a valuable predictive biomarker and therapeutic target. Current American Society of Clinical Oncology (ASCO) guidelines mandate that the ErbB status is evaluated in every invasive breast cancer to select the appropriate therapy, either at the time of diagnosis or recurrence [87,88]. Breast tumours overexpressing ErbB2 show a significant response to ErbB2 targeting agents such as trastuzumab or lapatinib [89,90,91,92]. However, in contrast to breast cancer, the treatment of ErbB2 overexpressing tumours of the endometrium and stomach did not yield comparable positive results [93,94]. Furthermore, activating mutations of ErbB2 were identified few years ago [95,96]. Immunohistochemistry and FISH demonstrated that most cancer cells containing such mutations were not overexpressing the ErbB2 protein. The afflicted cancer cells exhibited different sensitivities towards anti-cancer agents [95,96]. These findings have a profound impact on the clinical management of cancer. Tumours found to harbour ErbB2 mutations may display addiction to ErbB2 signalling and sensitivity towards ErbB2 tyrosine kinase inhibitors [97]. If the correlation between ErbB2 mutations and drug responsiveness is confirmed in prospective clinical trials the screening for ErbB2 mutations in breast tumours will be mandatory before starting the therapy.

In the last years, ErbB3 signalling has gained considerable attention in cancer research. ErbB3 predominantly forms a heterodimer with ErbB2 and was found to be critically involved in tumour initiation and progression and is now considered one of the most active signalling dimers of the ErbB family in cancer [98,99,100]. Consistent with the findings of other groups, we could recently identify two distinct breast cancer populations that expressed either high (~60% of tumours) or low (~40% of tumours) levels of ErbB3. Interestingly, the highest ErbB3 expression was detected in ErbB2-positive specimen. Increased co-expression of ErbB2 and ErbB3 might critically influence oncogenic signalling, oncogene addiction and thereby responsiveness to TKIs [99]. Somatic mutations in the ErbB3 gene occur in approximately 10% of colon and gastric cancers and increased activity may also be involved in melanoma formation [17,101]. Inhibition of ErbB3 with low molecular weight inhibitors is difficult enterprise, as ErbB3 is missing the kinase domain and TKIs are therefore non-effective. Alternatively, various ErbB3 targeting antibodies are currently under investigation which might block heterodimerization with other ErbB proteins [101,102].

The involvement of ErbB4 in carcinogenesis has been less well addressed so far [103,104]. Williams et al. showed that this gene is overexpressed in colon cancer and postulated that it might promote carcinogenesis [105]. Mutations of the ErbB4 gene are not very frequent but some activating alterations were described in the kinase (D931Y and K935I) as well as extracellular (Y285C and D595V) domain and were suggested as putative drug targets [106].

Taken together, many cases of oncoprotein addiction of the ErbB family have been identified in the last decades. These were instrumental for the development of targeted cancer therapies and useful as predictive biomarkers in the clinic. However, cancer is a complex and multi-faceted disease and acquired drug resistance as well as non-oncogene addiction and synthetic lethality suggest that combination therapies might be the most effective remedies in the future [107,108,109,110,111,112,113,114,115,116]. For example, it has been shown that c-MET can compensate for the loss of EGFR signalling [49,117]. Hence, the effective inhibition of both receptors by combination therapies might overcome the shortcomings of single treatments [118,119].

## 3. Identification and Molecular Characterization of ErbB Oncogene Addiction in Preclinical Cancer Models

**Figure 1:**
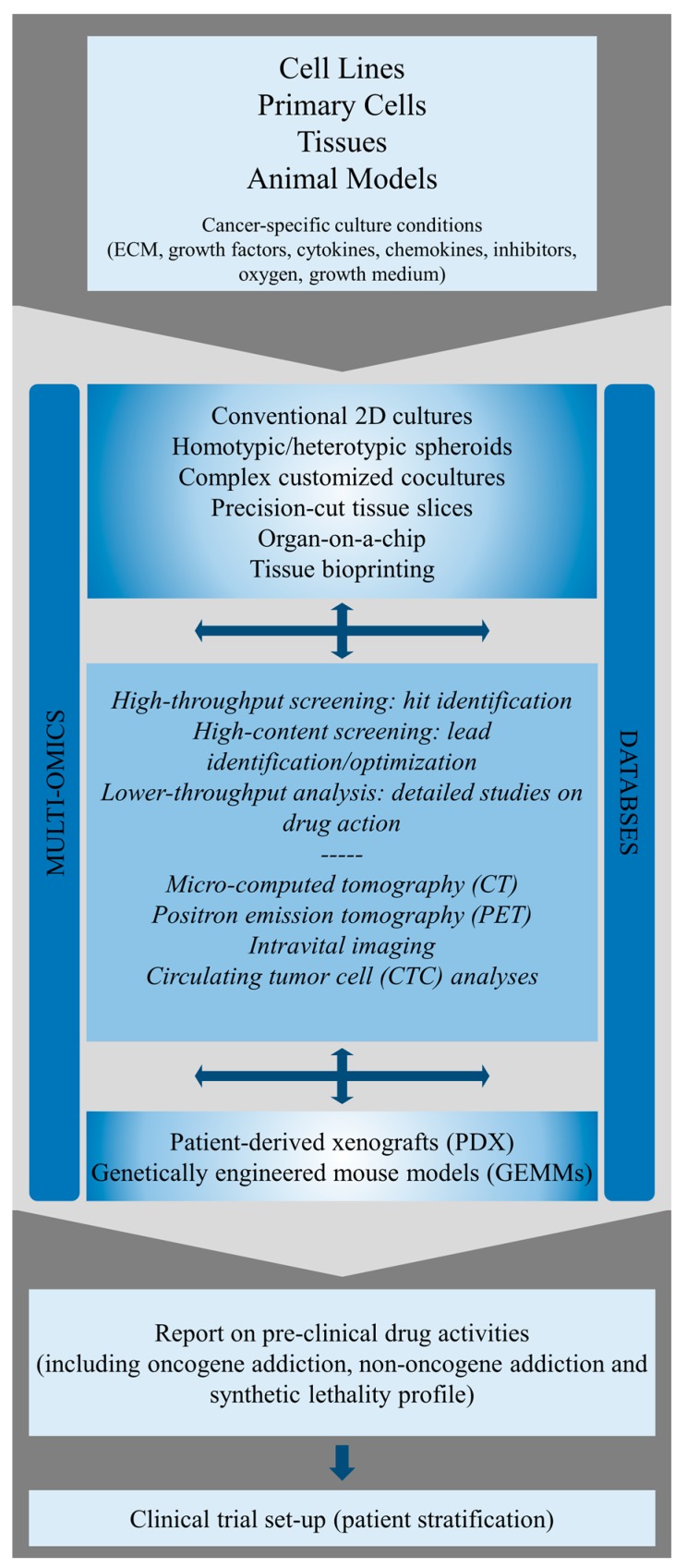
Multiparametric drug discovery for precision medicine. Conventional 2D cultures and complex 3D in vitro and in vivo models are obtained from various biological starting materials and animals (cell lines, primary cells, primary tissues, mice). Databases and multi-omics sciences (e.g., genomics, transcriptomics, proteomics, metabolomics) are used to select and establish physiological culture conditions for tissue engineering and disease modeling (e.g., EGF, FGF10, gastrin, RSPO1, Wnt3a for pancreatic cancer [137]) and to identify proper animal models. Multi-omics sciences, innovative drug testing technologies and bioimaging are instrumental for defining novel genotype-drug response relationships and determining drug efficacy. This will facilitate the appropriate design of clinical trials and reduce drug attrition rates.

There is a growing body of evidence that oncogene addiction may be present in a significant number of tumours in virtually all entities. Since the term was coined in 2002 by Bernard Weinstein, a large number of therapeutically relevant cases has been reported by many research groups [52,120]. However, the precise molecular mechanisms of this phenomenon including genetic streamlining, oncogenic shock and synthetic lethality still need to be characterized experimentally [57]. Therefore, it is of central importance to develop and characterize new in vitro and in vivo cancer models that allow for a more robust molecular characterization and validation of oncoprotein addiction and, more importantly, that facilitate the identification of new constellations of oncoprotein dependencies (see Figure 1). Several cell [79,121,122,123,124,125,126,127,128] and mouse models [103,125,127,129,130,131,132,133,134] have been successfully developed and used in the last 30 years for the analysis of ErbB-related oncoprotein dependencies. Despite steady advances in the field of tissue engineering, material sciences and cell culture technologies, monolayer-based cell culture models (2-dimensional, 2D) have mostly been the gold standard at early stages of preclinical drug development. 2D cell culture studies revealed that ErbB inhibition by TKIs could significantly reduce the survival of cell lines harbouring distinct ErbB mutations [135,136]. As described above, several EGFR mutations are known to render cancer cells hypersensitive (e.g., L858R, exon 19 deletions) or resistant (e.g., T790M) to targeted inhibitors. Many in vitro studies delineated not only the mode of action of ErbB inhibitors but also helped to better understand genotype-drug response relationships for guiding future drug design. 

Zhao and co-workers showed that several newly synthesized compounds effectively elicited cell death in the EGFR dependent NSCLC cell line HCC827 [138]. Recently the Lu group used the ErbB2 domain I-specific fully human antibody H2-18 to induce programmed cell death in the trastuzumab resistant breast cancer cell line HCC-1954 [136]. Due to the absence of a kinase domain, the ErbB3 receptor has been less appreciated as a potential drug target. However, its important role in cancer cell signalling, i.e., promoting tumour initiation and progression through PI3K/Akt signalling, has led to the development of highly potent small molecular weight compounds and monoclonal antibodies, which showed considerable efficacy in cancer cells in culture [8,139]. The recently developed multi-kinase inhibitor SKLB1206 showed promising results in inhibiting not only EGFR with gefitinib-sensitive and -resistant mutations but also showed considerable inhibition potency against ErbB2 and ErbB4 in cancer cell lines [140]. Importantly, several groups reported that c-MET mediates drug resistance after EGFR inhibition [118]. Focal amplification of the c-MET proto-oncogene rendered gefitinib-sensitive lung cancer cells resistant to TKIs. Strikingly, gefitinib sensitivity could be restored again upon c-MET pathway inhibition [118]. Moreover, c-Met was found to induce ErbB3 signalling in cancer cells and the inhibition of both, c-Met and EGFR caused cancer cell death [50]. Another report demonstrated that EGFR amplification and mutations in the PIK3CA gene are often acquired in response to erlotinib and PF00299804 treatment, which ultimately rendered them resistant to the EGFR inhibitor [141]. Apart from chemically synthesized compounds such as gefitinib or erlotinib, the metabolite butein, found in the stembark of cashews, was found to efficiently inhibit c-Met and EGFR and induce apoptosis in TKI-resistant cancer cells [46].

However, the highly artificial 2D culture conditions might not always reflect the actual drug efficacies in the clinic. Experimental culture set up (e.g., serum concentration, plastic dishes, substrate coatings) and the different strategies to assess drug sensitivity (MTS/MTT, CellTiter-Glo, alamarBlue) might lead to highly artificial preclinical results [46,78,142]. Hence, the predictability of 2D models has widely been questioned and there is an urgent need for models that much better predict drug efficacy and safety during early stages of drug discovery. Complex 3-dimensional (3D) in vitro models might represent a missing link between the non-physiological 2D cultures and the rather expensive animal models. In its simplest way, the 3D cell cultures are homotypic cell aggregates, commonly termed spheroids [143,144]. The generation of spheroids can be automatized and hence they are amenable to high-throughput phenotype-based drug discovery to study cell metabolism, proliferation, apoptosis, and migration [145,146,147,148,149,150]. Moreover, they might allow drug screening of anti-cancer agents in tailored microenvironments [151,152]. Several reports already demonstrated that the efficacy of drugs targeting ErbB family members often differed between 2D and 3D cancer models. Altogether, the reports suggested that the 3D models much better reflected the situation in the clinic. For instance, Pickl and Ries showed that ErbB signalling was fundamentally different between 2D and 3D cultures. When they treated ErbB2 overexpressing breast cancer cells with the ErbB2 targeting monoclonal antibody trastuzumab, the cell viability could be significantly reduced in the 3D cultures, whereas the 2D monolayers were only marginally affected. They could demonstrate that the distinct sensitivity to trastuzumab in 3D was based on differential ErbB2 heterodimerization and downstream signalling [153]. In concordance with that finding, the group of M.J. Bissell could clearly demonstrate that the response of ErbB2 overexpressing breast cancer cell lines to trastuzumab, pertuzumab and lapatinib was significantly different in 2D and 3D cultures [154]. 3D cultivation induced a switch of ErbB2 downstream signalling from PI3K/Akt to Ras-MAPK signalling. Another study investigated the drug response in 2D cultures and 3D neuroshperes, both derived from glioblastoma resections. When treated with the EGFR inhibitor erlotinib the 2D cells responded only weakly, whereas a strong growth inhibition was detected in neurospheres [155]. In addition, the same authors demonstrated that neurosphere initiation was strongly blocked by the simultaneous treatment with erlotinib and the hedgehog inhibitor cyclopamine. Furthermore, a recent study showed that NSCLC cell lines changed the cellular response to growth factors as well as inhibitors of EGFR signalling when cultivated as 3D spheroids, that better mimicked the natural tumour microenvironment [156]. In line with these findings, we could show that NSCLC cells expressing different EGFR mutants exhibited prominent EGFR oncoprotein addiction only in the 3D culture set up (unpublished results). Interestingly, in KRAS wild-type cell lines derived from colorectal cancer, inhibition of EGFR signalling was less effective in 3D cultures grown in laminin-rich extracellular matrices, emphasizing the influence of the ECM on cancer growth and drug response [157].

In the last decade, the tumour microenvironment (TME) was integrated into complex 3D cell models to more reliably assess and characterize oncoprotein networks [158,159,160]. This is of major importance, as the tumour stroma can occupy a large area within the whole tumour mass [161]. There is a reciprocal molecular crosstalk between the stromal and the cancer cells. Various growth- and survival-promoting factors, cytokines, and chemokines from the tumour stroma have been identified to drive cancer progression and metastasis [162,163,164,165,166,167]. Hence, customized heterotypic 3D co-cultures that incorporate various stromal compartments might facilitate the identification and molecular characterization of the effects of stromal cells on drug efficacy and resistance, and simultaneously allow the proper assessment of oncoprotein addiction and synthetic lethality [143,168,169]. For example, Rudisch et al. investigated the contribution of factors secreted by fibroblasts to cancer cell invasion [170]. The cytokine secretome revealed a cytokine fingerprint which pointed towards EMT induction. Interestingly, Byers and his group revealed that EMT gene signatures predicted resistance to EGFR and PI3K inhibitors [171]. At present, novel bioluminescent platforms (CS-BLI) allow the discrimination between tumour and stromal cell viability after drug exposure. Thereby it is possible to selectively quantify tumour cell viability in the presence versus absence of stromal cells [172].

Beside homotypic and heterotypic 3D spheroids, viable tissue explants directly derived from the primary tumour may serve as physiologically relevant ex vivo cancer models [173,174,175,176,177]. In contrast to spheroids, primary tissue explants exhibit virtually all cell types of a tumour, including the correct tumour-stroma ratio and the proper spatial distribution of the cells [175,178]. Therefore, they perfectly mimic the in vivo topology in cancer patients [177]. Cellular heterogeneity, tissue architecture and cell-cell communications are largely preserved in these precision-cut tumour slices (PCTS) [177]. Using (semi-) automated slicers, PCTS with well-defined diameter, thickness and high viability can be obtained from solid tumours and directly subjected to pharmacological studies [174,178,179]. PCTS can be continuously analysed during drug exposure using high content phenotype-based drug screening platforms [175,177]. This may allow a reproducible and standardized drug evaluation on native patient material. The new tissue engineering technologies make PCTS superior to spheroid cultures, especially when investigating the contribution of the tumour stroma to drug efficacy, (non-) oncoprotein addiction and synthetic lethality [177]. Prior to drug testing the RNA and DNA can be isolated from the patients’ tissue slices to determine the mutation status of specific biomarkers or perform large-scale genome or exome sequencing. On that basis, novel genotype-drug response relationships could be identified, that later facilitate appropriate patient stratification in clinical trials and help to discriminate between responders and non-responders [179,180,181,182,183]. In proof of principle studies, we could recently demonstrate that the drug sensitivity of PCTS derived from breast cancer specimen closely resembles the in vivo situation in cancer patients [184]. After treating PCTS with the selective EGFR/ErbB2 inhibitor lapatinib, we assessed the number of proliferating (Ki67) and apoptotic cells (cleaved and activated caspase-3) in situ using confocal immunofluorescence microscopy. Strikingly, only PCTS that expressed high levels of ErbB2 were sensitive to lapatinib treatment. The lapatinib treated PCTS exhibited an increase in the amount of apoptotic cells and concomitantly a decrease in cell proliferation [184].

Alternatively, PCTS could be used to generate patient-derived xenograft (PDX) mouse models [185]. PCTS or a larger fragment of a patient’s tumour is engrafted into mice without prior in vitro expansion, preserving to a considerable extent the architecture and physiology of the tumour in the mouse. Tumour evolution and adaptive response to therapy in vivo can be analysed in the PDX mice. A growing number of studies, many of which focused on ErbB signalling is emphasising the applicability of PDX for drug discovery [185]. A recent study found a direct correlation between high expression levels of EGFR and cetuximab activity in a PDX model of human NSCLC [186]. They concluded that high EGFR expression levels may be a predictive biomarker for the selection of NSCLC patients who might benefit from cetuximab treatment. Wu and co-workers established PDX models of oesophageal squamous cell carcinomas [187]. They could show significant tumour regression upon treatment with trastuzumab. However, drug efficacy was dependent on ErbB2 gene amplification and PIK3CA mutational status [187]. Another report provided evidence that the growth of head and neck squamous cell carcinomas (HNSCC) were reduced in PDX mice by dual targeting of EGFR and ErbB3 [188]. They revealed that cetuximab treatment induced the upregulation of ErbB3 as a compensatory resistance mechanism in their PDX model. In patient-derived gastric adenocarcinoma xenograft models, Chen et al. demonstrated the therapeutic effect of trastuzumab. The ErbB2 overexpressing model showed significant tumour regression upon treatment indicating the tumour’s addiction to ErbB2 signalling [189].

Finally, genetically engineered mouse models (GEMMS) have been used extensively in the characterization of drugs targeting ErbB family members [129,190,191,192]. In GEMMS, the tumours contain an in vivo tumour microenvironment comprising a multitude of stromal cells, an intact vasculature and an intact immune system [185]. GEMMs are designed to carry specific genetic lesions known to be essential for driving tumourigenesis, representing reasonable tools to validate novel genotype-drug response relationships. For that reason GEMMs and PDX models are often studied simultaneously to phase I/II trials to improve data interpretation in initial phases of clinical drug testing [185]. The group around Politi developed a transgenic mouse model expressing exon 19 mutated EGFR receptors under the control of doxycycline. These mutations are frequently found in human lung adenocarcinomas and are known to be essential for tumourigenesis. The group could show via magnetic resonance imaging and histopathology that doxycycline withdrawal or treatment with the EGFR inhibitor erlotinib caused rapid tumour regression [129].

Taken together, the studies on ErbB proteins clearly indicated that complex cell- and tissue-based models need to be integrated in preclinical drug testing. However, intense research efforts are still necessary to fully exploit the potential of such models for preclinical drug discovery.

## 4. Targeted ErbB Therapies and Evidence-Based Treatments in Precision Medicine

Based on the prominent role of ErbB signalling in cancer development and progression, ErbB receptors are promising molecules for targeted therapy. Two main approaches that are already implicated in the clinic are followed here. On the one side, monoclonal antibodies directed against the extracellular domains of the receptors can interfere with ligand binding and/or receptor dimerization, thereby inhibiting autophosphorylation and initiating internalization and degradation of the receptor. On the other hand, low molecular weight compounds, such as TKIs are developed. By binding to the intracellular tyrosine kinase domain (TKD) of the receptors, these drugs block the proliferative signalling cascades of specific ErbB family members.

Irrespective of the nature of the targeting compound (antibody or TKI), it is important to note that only those subgroups of patients will benefit from the targeted treatments, whose tumours are heavily dependent on dysregulated ErbB signalling. Therefore, patients have to be stratified according to the individual genetic composition of their tumours. Targeted therapies and evidence-based treatments are critical features of personalized medicine (precision medicine).

Gefitinib (Iressa^®^, AstraZeneca, London, UK) was the first EGFR specific TKI to be approved by the FDA for the treatment of NSCLCs after the clinical trial IDEAL I/II had shown an 18.4% (CI 95%) response rate [193]. However, approval was discontinued as the phase III study ISEL failed to show a beneficial effect of gefitinib in comparison to placebo treatment [194]. In 2004, the link between somatic mutations of EGFR and excellent response rates to EGFR-specific TKIs was identified [195,196]. Of particular relevance are L858R in exon 21 and small deletions in exon 19 that both lead to EGFR hyper-activation. Based on these observations patients carrying such sensitizing mutations were selected for further studies and response rates of 60–80%, along with a progression-free survival of approximately 10 months, were obtained [77,197]. Therefore, gefitinib and erlotinib (Tarceva^®^, Genentech, South San Francisco, CA, USA) were approved by the FDA for the treatment of EGFR-mutated NSCLCs. Both compounds reversibly bind to the ATP-binding pocket of the mutated EGFR-TKD thereby inhibiting EGFR tyrosine kinase activity. However, despite initial benefit, most tumours develop resistance to these drugs, which is in more than 50% of the patients mediated by an additional point mutation in EGFR exon 20. The so called T790M gatekeeper mutation results in an increased affinity of EGFR to ATP as compared to its affinity to first-generation TKIs. Therefore, in addition to sensitizing mutations, patients are screened for the presence of T790M prior to (further) treatment with gefitinib or erlotinib [80,198,199,200].

Drug resistance mediated by T790M has inspired the development of second-generation EGFR TKIs, which are supposed to bind to the EGFR-TKD in an irreversible manner [201]. Several drugs with these characteristics were tested in clinical trials, but most of them except for afatinib (Gilotrif^®^; Boehringer Ingelheim, Ingelheim, Germany) had failed due to either lack of clinical efficacy or due to limitations of clinical dosing because of toxicity. Toxicity of the second-generation EGFR-TKIs was mediated by non-selective binding to EGFR wildtype [202,203]. Selective inhibitors to EGFR mutants that also bind irreversibly to the TKD, would be a promising approach to overcome T790M-mediated resistance in NSCLC patients. Some third-generation EGFR inhibitors, such as osimertinib (Tagrisso^®^, AZD9291, AstraZeneca, London, UK), WZ4002, and rociletinib (CO-1686; Clovis Oncology, Inc., Bouler, CO, USA), have already been shown to work effectively against cell lines and murine models harbouring the T790M mutation while sparing EGFR wild type cells [204]. Osimertinib has been classified a breakthrough compound, as it shows 200-fold selectivity for T790M/L858R mutant EGFR over the wild type protein and has demonstrated perfect objective response rates in patients with T790M-positive NSCLC who had progressed on a first-generation EGFR TKI [205]. In 2015, the FDA approved osimertinib for the treatment of patients with metastatic T790M-positive NSCLC who had progressed on prior systemic therapy. However, mutations resulting in resistance to osimertinib have already been observed, and moreover, overexpression of ErbB2 and/or ErbB3 could also confer resistance to TKI treatment. Therefore, intense research activities are ongoing for TKIs that overcome the different resistance mechanisms [81].

Cetuximab (Erbitux^®^, Bristol-Myers Squibb, New York, NY, USA) and panitumumab (Vectibix^®^, Amgen, Thousand Oaks, CA, USA) are both monoclonal antibodies targeting EGFR with high affinity [206]. While cetuximab is a human/mouse chimeric antibody of the IgG1 class, panitumumab is a fully human IgG2 antibody. Both target the extracellular domain of the EGFR, thereby inhibiting intracellular signalling, proliferation and angiogenesis, as well as stimulating apoptosis and preventing metastasis [207,208]. Presently, the use of cetuximab or panitumumab in combination with chemotherapy is restricted to metastatic colorectal cancer (mCRC) patients with KRAS and NRAS wildtype, because it was observed that the antibodies had no effect in mCRC patients with activating mutations in the KRAS or NRAS oncogene [209,210]. Trials testing the effectiveness of anti-EGFR mABs in combination with chemotherapy in NSCLC patients are currently ongoing [211]. In vitro studies showed mAB binding also to mutant EGFR, and initial tests had demonstrated some promising effects [206]. However, recent studies demonstrated that acquired mutations within the ectodomain of the EGFR can cause resistance to cetuximab and/or panitumumab treatment [212,213,214,215,216]. Mixtures of two novel nonoverlapping anti-EGFR mABs (Sym004) effectively bound and abrogated ligand-induced phosphorylation of individual EGFR mutants that were resistant to cetuximab treatment [217]. Similarly, an oligoclonal antibody MM-151 that binds multiple regions of the EGFR extracellular domain inhibits EGFR signalling and cell proliferation in preclinical models, including patient-derived cells carrying mutant EGFR [218].

The role of ErbB2 overexpression has been most extensively studied in breast cancer, but has also been reported to play a role in other solid tumours, such as gastric cancer [219,220,221]. Similar to EGFR, ErbB2 targeting antibodies, i.e., trastuzumab (Herceptin™; Genentech) and pertuzumab (Perjeta^®^, Genentech), as well as TKIs such as lapatinib (Tykerb™, GlaxoSmithKline, Brentford, UK), were successfully used to treat ErbB2+ tumours. Trastuzumab, a humanized mAB targeting the extracellular domain of ErbB2, leads to the internalization and down-regulation of cell surface ErbB2 [222], inhibition of the PI3K/Akt pathway [223], cell cycle arrest, inhibition of angiogenesis [224] and antibody-dependent cell-meditated cytotoxicity (ADCC) [225,226]. However, de novo as well as acquired resistance against trastuzumab treatment has been reported for about half of the ErbB2+ cancers [227,228]. Resistance mechanisms against trastuzumab include alternative ways of activating the PI3K/Akt/mTOR pathway, expression of truncated, constitutively active versions of ErbB2, activation of IGFR signalling, overexpression of c-MET, SRC activation, or inhibition of the innate and adaptive immune system (reviewed in [229]).

Pertuzumab, also a humanized mAB, targets a different site of the extracellular ErbB2 domain, i.e., the dimerization arm, thereby blocking dimerization with other ErbB-family members and consequently inhibiting downstream mitogenic signalling [230]. Much less is known about resistance mechanisms against pertuzumab, but involvement of alternative ErbB heterodimers (e.g., EGFR/ErbB3) as well as specific activation of the PI3K/Akt/mTOR pathway have been observed in vitro [229].

Lapatinib is a first-generation reversible TKI targeting ErbB2 and additionally EGFR, ERK1/2 and Akt kinases [231]. Like TKIs targeting the EGFR, de novo as well as acquired resistance has been described for lapatinib. Resistance mechanisms include mutation of ErbB2, activation of the PI3K/Akt/mTOR pathway, activation of other RTKs, non-RTKs, autophagy, apoptosis, microRNA (miRNA), tumour metabolism, cell cycle, and heat shock protein (HSP) (reviewed by [70]). Novel irreversibly binding TKIs such as afatinib (anti-EGFR and -ErbB2) or neratinib (anti-EGFR, -ErbB2 and -ErbB4; Puma Biotechnology, Los Angeles, CA, USA) are currently evaluated, also with respect to overcome lapatinib resistance. Approaches to overcome ErbB2-targeting drug resistance in general, such as combinations of trastuzumab with pertuzumab, or with lapatinib, or combining lapatinib with PI3K inhibitors are also ongoing [229,232].

Due to its inactive TKD, ErbB3 was not thought to be important for cancer development or progression for a long time. Although it is still supposed not to be oncogenic on its own, ErbB3 is now considered a critical factor as a dimerization partner in a number of different cancers overexpressing ErbB2 or EGFR [233]. Moreover, ErbB3 has been reported to be a key player in the acquired and de novo resistance against ErbB-targeted therapies, such as cetuximab or trastuzumab, against chemotherapeutics, tamoxifen, anti-insulin-like growth factor 1 receptor (IGF-1R) therapies, as well as in castration resistance [234,235,236,237,238,239,240]. Consequently, ErbB3 is a promising therapeutic target, and the development of several ErbB3-targeting molecules including classical monospecific antibodies but also bispecific antibodies as well as alternative scaffolds is currently in progress [241]. The monoclonal fully human IgG1 patritumab (U3-1287; Amgen/Daiichi-Sankyo, Tokyo, Japan) was found using the XenoMouse^®^ technology and has been shown to have a 1–3 nM affinity to ErbB3 [242,243]. Initial results are promising as it was shown to inhibit basal as well as ligand induced signalling by inducing fast receptor internalization and degradation and blocking ErbB3 activation, respectively [244]. Of all anti-ErbB3 agents, patritumab is the one most advanced in clinical settings, being currently tested in a phase III trial for the treatment of NSCLC patients in combination with erlotinib [NCT02134015]. Other ErbB3-targeting molecules currently tested in clinical trials are excellently summarized in a recent review by Malm et al. [241].

Apart from ErbB3 emerging as an interesting therapeutic target, this ErbB-family member would also represent a valuable biomarker as overexpression of ErbB3 correlates with poor prognosis in different cancers such as breast, ovarian, lung and colon [245,246,247,248]. In addition to analysing ErbB3 expression levels, assessing the presence of the ErbB3-activating ligand Nrg would be of clinical interest, as increased levels of Nrg have been observed in several cancers, indicating autocrine signalling [249].

Reports on the role of ErbB4 in cancer are contradicting, suggesting pro- as well as anti-tumoural effects of ErbB4 depending on cancer subtypes and the ErbB4 isoform expressed [250]. Because of the controversial role of this ErbB-family member in cancer development, ErbB4 is so far not considered a validated therapeutic target, thus no specific ErbB4-targeting antibodies have been tested in clinical trials yet.

## 5. Conclusions and Outlook

Targeted therapies and companion biomarker diagnostics are powerful tools for precision medicine. Here we have summarized critical roles of ErbB family members in cancer development. ErbB proteins have been instrumental for understanding oncoprotein addiction, both in preclinical as well as in clinical settings. The roles of EGFR and ErbB2 in cancer development are presently well established, whereas data on ErbB3 and ErbB4 are still rather fragmentary. In the last decade, a diverse set of second and third generation ErbB targeting drugs (TKIs and antibodies) were developed that counteract de novo or acquired drug resistance. These drugs will further prolong the overall survival time of cancer patients experiencing an advanced disease.

In the future, heterotypic 3D in vitro models will be essential to fully understand the complex signalling circuits of oncoproteins and to identify novel genotype-drug response relationships, (non-) oncoprotein addictions and synthetic lethality. Such cancer models will be instrumental to accurately predict clinical drug efficacy and safety. In this regard, patient-derived xenograft (PDX) mouse models will allow to define drug efficacies in an in vivo environment. We think that in the future it will be imperative to exploit a vast array of preclinical cancer models in combination with large scale genomic and proteomic analyses and high-content phenotype-based drug discovery. Such a strategy will provide in-depth information on oncoprotein networks in cancer and yield novel insights of how the genotype might influence drug efficacy and toxicity. The complex cancer models will be instrumental for the appropriate validation of drugs and their corresponding targets on the preclinical level. This knowledge will be of utmost importance for the appropriate design and execution of clinical trials.
